# Artificial Intelligence in Medical Care – Patients' Perceptions on Caregiving Relationships and Ethics: A Qualitative Study

**DOI:** 10.1111/hex.70216

**Published:** 2025-03-17

**Authors:** Jana Gundlack, Sarah Negash, Carolin Thiel, Charlotte Buch, Jan Schildmann, Susanne Unverzagt, Rafael Mikolajczyk, Thomas Frese, PEAK consortium, Hans‐Ulrich Prokosch, Timo Apfelbacher, Carsten Fluck, Nadja Kartschmit, Iryna Manuilova, Jan Christoph

**Affiliations:** ^1^ Institute of General Practice & Family Medicine, Interdisciplinary Center of Health Sciences Medical Faculty of the Martin Luther University Halle‐Wittenberg Halle (Saale) Germany; ^2^ Institute for Medical Epidemiology, Biometrics and Informatics, Interdisciplinary Center for Health Sciences Medical Faculty of the Martin Luther University Halle‐Wittenberg Halle (Saale) Germany; ^3^ SRH University of Applied Health Sciences Heidelberg Germany; ^4^ Institute for History and Ethics of Medicine, Interdisciplinary Center for Health Sciences Medical Faculty of the Martin Luther University Halle‐Wittenberg Halle (Saale) Germany

**Keywords:** artificial intelligence, ethics, medical care, outpatients, patients, perceptions, relationship

## Abstract

**Introduction:**

Artificial intelligence (AI) offers several opportunities to enhance medical care, but practical application is limited. Consideration of patient needs is essential for the successful implementation of AI‐based systems. Few studies have explored patients' perceptions, especially in Germany, resulting in insufficient exploration of perspectives of outpatients, older patients and patients with chronic diseases. We aimed to explore how patients perceive AI in medical care, focusing on relationships to physicians and ethical aspects.

**Methods:**

We conducted a qualitative study with six semi‐structured focus groups from June 2022 to March 2023. We analysed data using a content analysis approach by systemising the textual material via a coding system. Participants were mostly recruited from outpatient settings in the regions of Halle and Erlangen, Germany. They were enrolled primarily through convenience sampling supplemented by purposive sampling.

**Results:**

Patients (*N* = 35; 13 females, 22 males) with a median age of 50 years participated. Participants were mixed in socioeconomic status and affinity for new technology. Most had chronic diseases. Perceived main advantages of AI were its efficient and flawless functioning, its ability to process and provide large data volume, and increased patient safety. Major perceived disadvantages were impersonality, potential data security issues, and fear of errors based on medical staff relying too much on AI. A dominant theme was that human interaction, personal conversation, and understanding of emotions cannot be replaced by AI. Participants emphasised the need to involve everyone in the informing process about AI. Most considered physicians as responsible for decisions resulting from AI applications. Transparency of data use and data protection were other important points.

**Conclusions:**

Patients could generally imagine AI as support in medical care if its usage is focused on patient well‐being and the human relationship is maintained. Including patients' needs in the development of AI and adequate communication about AI systems are essential for successful implementation in practice.

**Patient or Public Contribution:**

Patients' perceptions as participants in this study were crucial. Further, patients assessed the presentation and comprehensibility of the research material during a pretest, and recommended adaptations were implemented. After each FG, space was provided for requesting modifications and discussion.

**List of abbreviations**
AI: artificial intelligence
FG: focus group
GP: general practitioner
SES: socioeconomic status

## 1 Background

Artificial intelligence (AI) offers several opportunities to enhance medical care, also by addressing patients' needs [1, 2, 3]. AI‐based systems are already employed in various healthcare settings, including patient monitoring, diagnosis and treatment [4]. For instance, applications assist patients in lifestyle improvement [5] or are approved for evaluating patients' symptoms [6].

While a universal definition of AI is still lacking, we used a broad understanding of AI involving using machines to simulate human reasoning and intelligent behaviour, including thinking, learning, and reasoning, aiming to solve complex problems that previously could only be solved by human experts [7]. Subfields of AI include machine learning, deep learning, natural language processing, and computer vision [8].

Although some AI‐based systems are used in medical care, their potential is greater than their actual practical application. This hesitancy might be due to lacking quality and safety requirements for several devices, as well as end‐users' doubts about the accuracy and quality of the AI algorithm's output, leading to less trust [9, 10]. Additionally, there are many unresolved questions, e.g. regarding trust and accountability, privacy and data protection, autonomy and informed consent [11, 12, 13, 14, 15]. Another reason might be a lack of communication between developers and end‐users, which is essential for a responsible and successful development [16, 17]. As potential healthcare end‐users are healthcare professionals, including physicians and nurses, as well as patients, exploring and addressing their perspectives is essential for practical implementation. Patients have an increasing desire to participate in the healthcare process [18]. The service‐dominant logic suggests a shift from a provider perspective to a customer‐centred one, in which patients become co‐creators of care [19]. Involving patients (as well as healthcare professionals) not only as end‐users, but also as participants in the development process [20], and focusing on patient‐centred outcomes, plays an important role in the successful implementation of AI in healthcare, as the current perspective of the US Food and Drug Administration demonstrates [21].

Previous studies have mainly investigated the perceptions of patients in relation to specific applications or specialisms in medicine [22, 23] or have used a quantitative approach [24]. This is also evident in Germany, where there is a paucity of research on this subject [25, 26, 27, 28]. However, a qualitative approach is suitable to collect patients' perspectives [29], whose integration in a process can generate benefits [30]. Further, the perspectives of certain patient groups, such as outpatients, older patients, patients with chronic diseases, and those from lower socioeconomic backgrounds, have not been sufficiently explored [25, 31, 32].

This study aimed to explore how patients perceive AI use in medical care. This paper focuses on perceived advantages and disadvantages of AI, the perceived influence of AI in medical care on patients, the relationship between patient and physician, and ethical aspects.

## 2 Methods

### 2.1 Study Design and Participants

This qualitative study is part of the project “Perspectives on the Use and Acceptance of AI in Medical Care (PEAK)”, which examines perceptions among patients and physicians. We conducted semi‐structured focus groups (FGs) with participants primarily recruited from outpatient settings in the region of Halle and Erlangen, Germany, along with one FG of inpatients from a district hospital for psychological illnesses in Erlangen. Included were adults with first‐hand experience in the German healthcare system, excluding those under 18, lacking proficiency in German, or unable to consent. We contacted participants through study information leaflets and direct approach by telephone and face‐to‐face, also with the help of facility staff. Recruitment primarily employed convenience sampling supplemented by purposive sampling to enhance diversity in sociodemographic characteristics and healthcare experiences, added by snowball sampling. Participants provided written informed consent, were informed of their rights, and participated voluntarily without financial compensation. The local ethics committee approved the study protocol.

### 2.2 Development of Topic Guide and Application Examples

We developed a topic guide using literature from a PubMed search and guided by Helfferich's methods [33] and Krueger and Casey's approach to FG guides [34]. The guide featured open‐ended questions to explore patients' perceptions of AI in healthcare, including its advantages and disadvantages, possible influence on physician–patient relationships and on patients, and related ethical aspects (see Supporting Information: File S1). As a thematic introduction to the FGs, we created a video defining AI and showcasing three potential healthcare applications: (a) diagnosis: symptom check via Ada app [6, 35], (b) treatment: alternative medication plan [36], and (c) process optimisation: documentation assistance (via voice recognition) [37]. We varied the example sequence across groups to reduce bias, pretesting all materials.

### 2.3 Data Collection

We gathered participants' sociodemographic and technology affinity data, the latter assessed using the Subjective Technology Adaptive Inventory questionnaire's perceived technology competence scale [38]. We conducted six FGs between June 23, 2022, and March 1, 2023, at university or medical practice locations, including five to eight participants each. Sessions began with the video introduction, followed by discussions prompted by the FG guide. The interviewer was unfamiliar to most participants and gained experience in conducting FGs by testing the research material with colleagues and patients. We ensured that all participants had an opportunity to express their own opinions. We audio‐recorded all FGs, with team members also taking field notes, continuing until thematic saturation was reached.

### 2.4 Data Analysis

Using a content analysis approach [39], we systematically analysed the textual material using a coding system to extract patients' perceptions and expectations of the topic. Following the content‐analytic communication model, the analysis aimed to extrapolate insights about the subject matter and the cognitive background of the communicators. After transcription, FGs were analysed using MAXQDA 2022. We maintained anonymity by replacing names with pseudonyms. We collaboratively developed a coding system by independently coding a representative FG, discussing, and refining codes until consensus. Two researchers applied the coding system to all FGs, discussing modifications after each FG until consensus. We generated main themes based on the FG guide using a deductive approach while identifying subthemes inductively derived from the data. We ensured validity and reliability during analysis [39] with homogeneous coded passages demonstrating an appropriate coding system and code definitions. Construct validity was confirmed through literature comparison. For reporting the data, we used guidance of the Standards for Reporting Qualitative Research [40].

## 3 Results

The six FGs lasted between 86 and 134 min with 35 participants total (13 females, 22 males). Median participant age was 50 years, ranging from 23 to 92 years, and most had chronic diseases (Table 1). The composition of groups differed regarding age, gender and health condition. Participants' socioeconomic status (SES) was mixed with a tendency toward higher SES. Most participants had a good relationship with their general practitioner (GP) and consulted them once or none in the prior 3 months. Participants differed regarding affinity for new technology with a slight tendency toward high and medium affinity. An additional table shows FGs' characteristics (see Supporting Information: File S2). A few patients reported already experiencing AI in medical care, while a third reported no experience.

**Table 1:** Participants' Demographics and Characteristics (*N* = 35).

Variable	*N* (%)
Age: Median 50.0 years, range 23–92 years	
Gender	
Female	13 (37)
Male	22 (63)
Highest education level	
General qualification for university entrance (12–13 years)	19 (54)
General certificate of secondary education (9*–*10 years)^a^	16 (46)
Vocational qualification^b^	
Completed vocational training	21 (60)
In vocational training	3 (8)
Advanced technical college certificate/university degree	15 (43)
No vocational qualification	2 (6)
Other vocational qualification	2 (6)
Employment status	
Employed^c^, ^d^	19 (54)
Not employed	15 (43)
Thereof pensioners	10 (29)
Thereof students	2 (6)
Chronic disease(s)^e^	
Yes	20 (57)
No	14 (40)
Frequency of GP consultation^e^	
Less than once every 3 months	15 (43)
Once every 3 months	13 (37)
Two to three times in 3 months	4 (11)
Four times or more in 3 months	2 (6)
Relationship to GP	
Very good	18 (51)
Rather good	13 (37)
Neutral	1 (3)
Rather poor	2 (6)
Very poor	1 (3)
Affinity for new technology^f^	
Low	7 (20)
Medium	12 (34)
High	16 (46)

Abbreviations: GP, general practitioner; NA, not applicable.

^a^ Includes the German ‘Hauptschulabschluss’.

^b^ Partially more than one vocational qualification existing.

^c^ Two participants simultaneously in vocational training.

^d^ One participant simultaneously in retirement.

^e^ One participant did not respond.

^f^ Scale: ≤ 2 low, > 2 to < 4 medium, ≥ 4 high.

### 3.1 Perceived Advantages of AI

Participants noted AI's accuracy and efficacy as primary advantages, and its ability to process and provide large amounts of relevant information as beneficial. They appreciated AI's potential to reduce errors and improve patient safety, e.g., by reducing medical side effects. Further perceived benefits were time savings and reduced workload for medical staff, which could reduce treatment times and save time for patient care. The impartiality of AI for unbiased treatment and its potential for neutral and simplified conversation was seen as beneficial. Other advantages included financial savings and shortened diagnostic paths (Table 2).

**Table 2:** Representative quotes about perceived advantages and disadvantages of AI with subthemes.

Codes	Representative quotes
**Perceived advantages of AI**	
*Accuracy and effectiveness*	‘I think they are sometimes, if you break it down to the program, perhaps much more efficient, quicker, make fewer mistakes if they have the right data sets’. (FG5‐P.3, 28 y)
*Processing and provision of relevant information*	‘The AI system has the following advantage. It has a large potential of basic knowledge of the same or similar events that can be evaluated. To be able to visualize an optimal solution, humans can't do that, yes’. (FG1‐P.1, 74 y)
*Low fallibility*	‘Perhaps this could prevent doctors' errors. Doctors are humans too. And they make mistakes too. And I think that this might be supportive’. (FG2‐P.1, 72 y)
*Increase in patient safety*	‘Well, I think that if AI takes over the entire medical care and organization of a patient, in any case there won't be, let's say, a paradoxical effect of medication. Because if we take several physicians. The GP knows nothing about the specialist; the specialist knows nothing about the GP. He doesn't know what the other one is prescribing. He prescribes this, paradoxical effect. I would say that this is largely absent in AI. Because he takes over the entire medical care. There is no more specialist, no more GP’. (FG5‐P.4, 52 y)
*Time savings for medical staff*	‘So, I don't think it matters in which area you use it, it is definitely a time saver…. Yes, like especially for the medical staff’. (FG4‐P.1, 24 y)
*Reduced workload/content‐related support for medical staff*	‘So, I also imagine it as a support and quality assurance at work, of a physician for example. For example, a radiologist. When he goes through the X‐ray images, that something might be pointed out: “That looks suspicious, for example. One should take a closer look at that.”…So, for example, that you can see another perspective that perhaps the physician doesn't have at the moment. And that you could compare the two, for example’. (FG4‐P.2, 24 y)
*Impartiality*	‘I: The computer, the AI wouldn't want to do it like that? FG5‐P.4: Exactly, that's right. Because they like don't have any profit motives. Because it just works by the book’. (FG5‐P.4, 52 y)
*Patient and neutral conversation*	‘And especially when it comes to communication. An AI doesn't get impatient either. You can ask it again and again what something means. They never get tired of explaining it again’. (FG3‐P.4, 23 y)
*Financial savings*	‘Well, also a financial saving then in terms of travel costs or medication costs or whatever’. (FG‐P1, 46 y)
*Shortened diagnostic paths*	‘The main problem we face is exactly what you're saying, you actually have this huge time window between a diagnosis and further treatment by a specialist. If I shorten all that because I have access to the data, because I have access to the appointment scheduling, yes? Then of course it's a short path’. (FG1‐P.5, 62 y)
**Perceived disadvantages of AI**	
*Impersonality*	‘The disadvantage would be, I mean, to some extent, that you're just some number. Well, I need my doctor. Face to face. So, going to the doctor or via the internet and stuff like that, that's nothing. Human being has to remain the focus. Despite all the progress’. (FG1‐P.4, 70 y)
*Data security issues*	‘And in the end, AI is also a computer program, yes…. Isn't it also dangerous, so to speak, to use it in medicine, for example? Because I mean, every electronic item is hackable in some way’. (FG5‐P.3, 28 y)
*Medical staff rely too much on AI*	‘Where I see a bit of a danger is, let me say, that a doctor relies too much on the program. … But, for example, the doctor relies too much on the AI and overlooks something. Or, as I said, someone sneaks in and somehow deletes a data record. Or wants to make sure, for example, that a certain combination of medication is used, which then causes heart problems or something like that. And the doctor simply signs because he relies so much on the program, because it's just so reliable. Simply signed off without checking. And I think that's a very big danger, especially at the moment with the lack of staff in hospitals and nursing homes’. (FG5‐P.6, 29 y)
*AI's dependence on suitable data and humans*	‘It really is data records, and if these data records can somehow be manipulated, then the AI is somehow stuck. Because it just can't think outside the box, yes. Instead, it only has this data that it receives’. (FG5‐P6, 29 y)
*AI replaces human labour*	‘People are being replaced by machines. That's the thing, of course’. (FG5‐P.5, 68 y)
*Opacity of AI as a Black Box*	‘I think that's a basic problem of quality assurance, I would say. Because the very powerful systems are black boxes per se. You've got ‐. It just calculates a conditional expected value from a huge amount of training data. Which is not an explanation anywhere…. But if you now want to know why exactly it did this, you'll run into problems. Because the calculation process itself is basically a black box. You can't look inside, so to say’. (FG4‐P.5, 55 y)

Abbreviations: AI, artificial intelligence; y, years.

### 3.2 Perceived Disadvantages of AI

The primary drawback noted was AI's impersonality, citing its lack of personal connection, empathetic communication, and individualised approach. Participants were concerned over its inability to capture nonverbal cues or provide emotional support, risking the loss of personal touch in healthcare. Data security issues and potential over‐reliance on AI by medical staff, potentially leading to errors, as well as AI's dependence on data quality and humans, were also the main concerns. Other perceived disadvantages included potential labour losses and the opacity of complex AI systems (Table 2).

### 3.3 Potential Influence on Patients and the Relationship to the Physician

According to participants, AI's potential influence on the patient‐physician relationship would depend on appropriate integration, clear explanations and trust. However, a few participants considered AI as irrelevant in their treatment and expressed concerns about the level of involvement. Most patients preferred a personal component in medical treatment, which AI could not guarantee. They did not anticipate changes in their relationship with physicians or perceived AI as enriching as long as personal contact is maintained. Some considered AI secondary to treatment and recovery, trusting in their physician's judgement.

Participants discussed the need for greater technical awareness and openness due to relentless advancements in healthcare technology as well as caution and questioning of new applications’ goals (Table 3). Some argued that using AI on their own would be insufficient due to missing medical expertise, suggesting physicians should primarily use these systems. The decision to engage with AI was regarded as an individual choice.

**Table 3:** Representative quotes about potential influence on the physician–patient relationship and patients with subthemes.

Codes	Representative quotes
**Potential influence on the physician–patient relationship**	‘Ultimately, this system is actually an aid for the doctor. A tool. Whether he uses a scalpel or such an AI system, it actually only creates the possibility to carry out the analysis activity perhaps even more profoundly, right? To achieve a more concrete and helpful result’. (FG1‐P.1, 74 y)
**Potential influence on patients**	‘Well, one is not against it in principle, not by any stretch of the imagination, but as I said, a little caution is probably appropriate, I think. At least think about it in the end. Maybe look it up and read about it at the end, what's the point? What does it really mean in the end? One should do that. But to reject it on principle or something, no. I don't think that's going to help at all’. (FG1‐P.2, 73 y)
*Question of habituation and generation*	‘So, I think to myself, well, I also think it's a gradual process, that it's somehow just slowly coming into the physician's visit, where you might not even notice it…. And then you just accept it because you gradually grow into it’. (FG‐P1, 46 y)
	‘But I think that artificial intelligence is also difficult for some generations. Especially when patients have to take action themselves, with apps or whatever… So, I don't think it's equitable in that regard. I think the younger generation deals with it very differently to the older generation’. (FG‐P4, 37 y)
*AI is a vision of future*	‘I just know the daily routine, how we do it now, how we make the company goods. How we do all the project planning. And then always the visions. And there's still a huge gap in between. So, there's no bridge yet’. (FG4‐P.7, 56 y)
*Diverging diagnoses of AI and physician*	‘Well, I think the doctor should also question himself and should also question the AI and, if necessary, there could be a second opinion from another doctor. Let's see what he says’. (FG‐P2, 50 y)
Physician checks diagnosis/additional examinations	‘Or I could ask both of them first, that they‐. Or I probably don't ask them directly, but they would probably both check their results again. So, the AI simply the same data again. And see if the same output comes out. And the doctor will probably take her books and check the whole thing again’. (FG3‐P.4, 23 y)
Need for discussion	‘Well, you would have to communicate with each other again why the AI comes to this diagnosis and why the doctor sees it quite differently. Yes. That would have to be discussed somehow. Yes. Or‐. I mean, if the doctor can then explain that the variant which the AI has practically said is better for me, then I think if he explains it to me properly, I would accept it. Yes. But I think it really needs to be talked about. A lot of communicating’. (FG‐P1, 46 y)
Physician safety in decision‐making	‘And if that is conclusive and he can present it properly when he says: “For such and such a reason. Because, for example, these and these values have not been taken into account or the programming is poor.”, or, or, or. Why should I question that? He is a doctor for a reason…’. (FG1‐P.5, 62 y)
Collaboration between AI and humans	‘And then perhaps there is a more optimal solution after all, because both opinions somehow result in an average’. (FG1‐P.1, 74 y)
*Trust*	‘So that can be good or bad. But of course, it can always lead to a more complicated relationship between doctor and patient. Because I no longer only trust the doctor, which is also a good thing. But of course, it can also complicate things’. (FG4‐P.3, 30 y)
Trust in physicians	‘So of course I would trust the doctor. But I would also somehow trust the AI and get a third opinion. But you should not forget that these are professionals. They consult with several doctors. And they don't just say: “Yes, you have a tumor” if it's not even certain. They check with various other doctors, specialists. And with different laboratory parameters or whatever. That is why, if in doubt, I would actually tend to believe the group of doctors. Of course, I would not rule out the AI and would investigate further to see if that might also be taken into consideration. But I would tend to believe the doctors’. (FG4‐P.1, 24 y)
Uncertainty/depending on situation	‘If we get both results, then probably in the individual case, depending on what probability the AI spits out. And how certain the doctor is. So, I think it's difficult to give a general answer. Unless you have a fundamental rejection of AIs or something. Which I don't have. So, I would ‐, believe both of them. Depending on what gives me the better argument, so to speak. Yes’. (FG3‐P.6, 25 y)
Trust in AI	‘Yes. As we said, if the system works properly, then it is all clearly there. And my best doctor can also miss something. Or completely different developments have come out in the meantime. My doctor is not there yet. And the AI is already there. And that's why the AI’. (FG2‐P.2, 71 y)
*Irreplaceability of Caregivers*	‘A doctor–patient relationship is also always a relationship. And that can't be replaced’. (FG3‐P.5, 45 y)

Abbreviations: AI, artificial intelligence; y, years.

For this main theme, four subthemes arose (Table 3).

#### 3.3.1 Question of Habituation and Generation

AI's integration into medical care was expected to be gradual, with varying adaptability across generations, challenging older people more than younger ones. Some felt that this had more to do with individual technical disinterest than age. Patients expected that AI will play an increasingly important role in healthcare in the future as all generations become more technologically savvy. Participants' opinions on the timeframe for this development varied from 2 to 10 years to 30–100 years, mentioning that AI development is still in its infancy today, especially in medicine (excluding image recognition). Trust in AI was anticipated to grow, potentially altering the emotional dynamics of patient–physician interactions.

#### 3.3.2 Diverging Diagnoses of AI and Physician

Participants' reactions to different diagnoses from physicians and AI varied based on the physician‐patient relationship, situation, condition being treated, and personal preferences. One reaction on this situation mentioned by participants was to seek a third opinion from a human in case of discrepancies without clear explanations or in case of serious illnesses. Another was that physicians should verify different diagnoses with additional tests. Some patients emphasised the importance of clear explanations and mutual discussion, while others advocated for collaborative approaches between AI and humans. Some participants mentioned no doubt in physicians' diagnosis given physicians' confidence in own decisions. Participants highlighted that they should not have to make decisions themselves in case of conflicting diagnoses.

#### 3.3.3 Trust

Participants argued about the potential complication of the patient‐physician relationship due to an increasing role of AI in diagnosis, as it could create uncertainty about who to trust and the physician's competence. In case of diverging diagnoses, participants mentioned trust in physicians based on interpersonal skills, physicians' knowledge of patients' history, and the ability to comprehend human decision‐making. They also expressed uncertainties, with trust depending on physicians' coherent communication and expertise, database, AI's performance and its calculated probabilities, but also personal preferences. A few participants would trust AI, provided it works properly, and learning data are representative, as AI offers objectivity and access to current information.

#### 3.3.4 Irreplaceability of Caregivers

Participants mentioned that humans and personal interaction are essential and therefore not replaceable in healthcare, especially when it comes to personal conversation, emotional support, and vital decisions. They considered that loss of interaction could lead to loneliness, especially in care.

### 3.4 Ethical Aspects

#### 3.4.1 Patient Information

Opinions were divided on whether and how much information patients should receive about AI systems used in their treatment. Opinions ranged from expecting clear explanations regarding AI's functionality, safety, benefits, and drawbacks, to feeling that informed consent would be unnecessary if the outcomes were justified and sound. The need for comprehensive information across all age groups was emphasised, though some were indifferent or unsure about the extent of necessary details (Table 4).

**Table 4:** Representative quotes about ethical aspects with subthemes.

Codes	Representative quotes
*Patient Information*	
Important (for all age groups)	‘I think some basic information is not bad at all… like when you come into contact with new technology, that you at least get some information: A, what is it? B, what do I do with it? And what do you have to prepare for?’ (FG1‐P.5, 62 y)
Not important/unclear when information starts	‘Lastly… where do you draw the line again as to whether AI has been used or so? …Or where does it become relevant?’ (FG3‐P.4, 23 y)
*Responsibility*	
Developers	‘The developer must be the one who builds the system in such a way that A the information is collected and made available, and on the other hand, of course, the system is also protected. That it cannot be misused’. (FG1‐P.5, 62 y)
Physicians	‘But would there be any responsibility for the incorrect diagnosis?’ (FG3‐P.1, 28 y) ‘With the doctor then’. (FG3‐P.6, 25 y) ‘Yes’. (FG3‐P.4, 23 y)
Patients	‘With patients, I see it with honesty and openness’. (FG5‐P.4, 52 y)
*Privacy*	
Data protection important	‘So, it's also a question of security. So how safe is this system? That means this AI has to play a certain security role. And this is important data that comes in. And it has to be secured…. I think that's very important’. (FG3‐P.3, 78 y)
Data Usage AI‐unspecific	‘I think that most security risks are probably not caused by the use of AI itself. It's more likely to come from electronic data collection. Yes’. (FG3‐P.1, 28 y)
Data protection not important	‘But I would like to say one thing. Even 50 or 60 years ago, nobody knew what data protection was. We were treated. We got well. We were treated sensibly. And we weren't interested in what is called data protection today. I mean, sometimes it has to be passed on…. That's why I say it's all a bit bureaucratic, you can't say that’. (FG2‐P.5, 92 y)
*Comprehensibility of AI*	
Important for patients	‘I also think it's absolutely important, especially this comprehensibility. So yes, the AI ‐, it is absolutely important that I am also informed about how the AI did that. Why, basically, it came to its diagnosis, its result’. (FG4‐P.8, 30 y)
Not important for patients	‘So, I think it's not necessarily important for me to understand how this was achieved. But that enough data was used, that this probability is somehow based on a large amount of data, that he's right. But it doesn't matter how exactly it works that out now’. (FG3‐P.5, 45 y)
Patients as laypersons for AI and medicine	‘But I can't even grasp the details. It takes specialists to do that’. (FG1‐P.1, 74 y)
Sources available on request	‘And it would be important to me, for example, in case I ever have a question that a source can be given, for example. But there, for example, a statistic or something would be enough’. (FG3‐P.6, 25 y)
Expected by physicians/important for physicians	‘Yes, that would be very important to me now, to be honest. Because then we would be back to the error conspicuousness. I think the doctor has to be able to comprehend it, to be able to say whether what it says makes sense. And whether it is somehow realistic. To also determine if it might not be true in some way. So, I wouldn't have much confidence if the doctor told me: “The AI says that. I don't know why, but it will fit.” To exaggerate’. (FG4‐P.3, 30 y)
Not expected by physicians	‘Why should a doctor understand what the AI is like? I slap him with an algorithm or something and he shrugs his shoulders. He has no idea what it is. He just doesn't know. He cannot know. Why should he? He's a doctor of medicine. You're a graduate engineer or computer scientist. And you know it in computer science, but not in the medical field. It's the same with knowledge. He doesn't need to know’. (FG4‐P.6, 50 y)

*Abbreviations:* AI, Artificial Intelligence; y, years.

#### 3.4.2 Responsibility

Determining responsibility for AI's use in healthcare proved complex. Views ranged from end‐user responsibility to a tiered model shifting from developers to physicians and patients, with some advocating for shared responsibilities among all parties, including regulators. Developers were seen as responsible for ensuring AI's functionality and security, while physicians were viewed as responsible for AI ‐ primarily for applying AI judiciously, selecting a suitable system, and explaining its outputs to patients. Physicians were seen as mediators between the needs of patients and the capabilities of developers. Patients' responsibilities, when acknowledged, included engaging with their treatment, deciding on data disclosure, providing an accurate medical history, and, in independent use, following AI's recommendations (Table 4). Some participants did not believe patients held any responsibility.

#### 3.4.3 Privacy

Concerns about privacy focused on unauthorised data access and potential data misuse, contrasting with worries that excessive data protection could hinder AI's development. Transparency was deemed vital for managing treatment data access, particularly for sensitive conditions such as mental illnesses. Opinions ranged from data protection being important in relation to AI, to seeing no change in privacy or data protection due to AI. Some argued that security issues were not AI specific, and electronic data was more problematic. A minor aspect was that stringent consent requirements for data sharing would be obstructive, suggesting that data should be shareable among all stakeholders involved in treatment if it benefits patient recovery (Table 4).

#### 3.4.4 Comprehensibility of AI

Participants were split on the importance of understanding AI decisions. Some found comprehensibility difficult due to lack of medical and technical knowledge. While some preferred the ability to inquire about AI's data sources, others were less concerned, trusting in physicians' judgement and regulatory standards. Those valuing comprehensibility stressed the need for brief explanations of AI's basic functions and decision‐making processes, increasing understanding of treatment, and trust in AI. Participants stated physicians should comprehend the general functions and handling of the AI systems used, their data basis, and be able to interpret AI results. However, some believed that complete technical understanding might require specialist intervention, suggesting that a basic grasp by physicians might suffice (Table 4).

**Figure 1:**
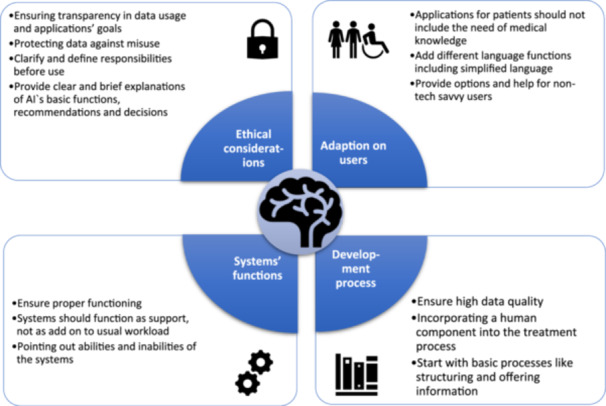
Recommendations for practical use/development of AI systems derived from patients' perceptions.

To help formulate recommendations for the practical implementation and development of AI systems, we summarised patients' perceptions in Figure 1.

## 4 Discussion

Our study underscored that while participants recognised the efficacy of AI, they firmly believed it cannot replace the essential human elements of empathy, personal interaction, and emotional understanding. Most participants placed greater trust in physicians over AI, particularly in scenarios of conflicting diagnoses. However, with ongoing development and increased familiarity with AI, this may change. Inclusive involvement of all individuals in the information process and patients' needs were deemed crucial to foster understanding and usage of AI. Responsibility for AI's application was a difficult issue, with no general consensus among participants and different responsibilities considered for developers, physicians, and patients. According to participants, ensuring transparency in data usage and protecting data against misuse were crucial.

Our findings resonate with those reported by Young et al. discussing AI's advantages and disadvantages [31], and Bindra et al., who mentioned AI's potential to improve healthcare, while also highlighting concerns about legal and ethical issues, human interaction, and data privacy [4]. This corresponds to the current perspectives of Kazakh patients, which support our findings [41]. AI could improve the efficiency and cost of healthcare and speed up diagnosis and treatment, though concerns remain about the loss of the human touch, privacy and other ethical and legal issues. Participants acknowledged AI's potential to improve patients' safety by reducing errors, suggesting its support in follow‐up. For example, AI chatbot interventions can support follow‐up and improve chronic disease management, as they are easily accessible, well accepted and comparable in effectiveness to interventions delivered by healthcare professionals, although challenges such as technical efficacy and issues and adequate training of AI need to be addressed [42]. This is consistent with a review of AI applications for children and adolescents that have the potential to improve chronic disease management, particularly in mental health, highlighting the need for clinical implementation to validate effectiveness in real‐world scenarios and ethical considerations [43]. Controversial to our findings Tursynbek et al. and Tran et al. state a more personalised care as perceived advantage of AI [41, 44]. McCradden's notion of ‘hopes and fears’ captures the ambivalent sentiments patients hold towards AI, given their limited prior exposure to the technology [45]. The general lack of awareness of specific AI applications [17] and concerns about the loss of the human touch are in line with the American public [46] and other healthcare stakeholders, such as nurses [47]. Although healthcare professionals are generally open to the use of AI as long as it reduces their workload and they are trained, their concerns about AI control and trust, ethical and regulatory issues, and maintaining compassionate care need to be addressed [47, 48, 49, 50, 51].

Most participants perceived AI as a vision of future that is just in its infancy, especially in medicine (except image recognition). This view can be embedded in current literature [52].

Several other studies highlight the importance of maintaining a personal relationship with personal conversation and emotional support [22, 41, 53]. Participants' opinion that AI cannot replace medical staff, due to the essential nature of personal interaction, corroborates other research findings [41, 54, 55, 56]. However, some studies suggest AI may eventually replace physicians [57, 58]. The question arises: Where does personal interaction actually begin? Participants struggled to define personality boundaries and primarily discussed facial expressions, gestures, nuances, and emotions, which seem essential in interpersonal relations. Despite the Computers Are Social Actors theory claiming ‘that the human–computer‐relationship is fundamentally social’ [59], participants perceived technical systems as inadequate in providing personal or interpersonal aspects. Also, Turkle claims that AI seems capable of intelligence, but not of real emotions [60]. Empathy, which is not exclusively based on algorithmic processes, cannot or should not be generated artificially [55].

Participants' uncertainty about whom to trust – physician or AI – agrees with current research [53]. However, most participants tended to trust physicians due to the interpersonal component and the ability to comprehend human decisions, aligning with results from a previous review [31]. This suggests trust in AI depends on comprehension and can be enhanced by providing information about its internal status and development sources [61]. Trustworthy AI must be transparent, comply with ethical and legal regulations, and be technically and socially feasible [62, 63]. In contrast, AI cannot be trustworthy by lacking requirements of trust such as emotional motives or accountability [64]. This may explain why most participants would preferentially trust the physician. These principles of trustworthy AI also align with the perceptions of healthcare professionals, highlighting the need for ethical and legal regulations (to protect patient welfare) and the preservation of the human touch [50, 65].

Some participants emphasised the importance of information and comprehensibility for understanding AI, which concurs with other research and must be considered in developing and implementing AI in healthcare [23, 32, 66, 67]. Despite not all included participants favoured being informed about AI usage, the importance to involve all individuals in the information process was emphasised, aligning with Ongena et al.'s findings on patients' general desire for thorough information about medical diagnostics [68]. Doing so might be beneficial as many participants in our study admitted to having limited knowledge about AI and struggled to distinguish it from other digital devices. However, early engagement with patients, to allow their perspectives to inform the development process [17, 69] and providing information adapted to patient needs through co‐creation as value‐in‐use are essential for successful implementation [30]. Conversely, other participants argued that comprehensibility is unattainable for patients as laypersons or due to the opaque nature of AI, echoing findings from other studies [70]. Although participants believed that physicians, as end‐users, should understand the basic functions of AI, this may not be feasible as technical laypersons, leading to a lack of understanding of AI decisions [71]. While Tursynbek et al. showed that patients considered physicians to have a duty to provide easily accessible and understandable information about AI [41], our participants also mentioned the media and university facilities. Physicians must be aware of the functions and pitfalls of AI systems and be transparent about them to facilitate patients' informed consent [72, 73]. This empowers patients to participate in decision‐making and self‐management [41], but there are practical challenges to overcome in implementation [74].

While most of our participants viewed physicians as primarily responsible as end‐users, uncertainty prevailed on this matter [71] with participants' opinions being heterogenous [22]. Establishing a standard for responsibilities would be beneficial, as other findings also suggest [75]. Participants emphasised a responsible use of sensitive health information and the necessity to protect it [76], with a preference for concise but thorough privacy explanations [45]. The commitment to transparency, data protection and clarification of responsibilities along the AI lifecycle is also addressed in the recently enacted European AI Act, demonstrating the importance of addressing these patient concerns [77]. Participants' concern of data misuse aligns with other findings [45, 78], not only in context of AI [79], as some participants discussed the electronic recording of data as a security concern [62]. A discussion about AI and data might be more general about data usage for digital technologies [80], with the need for transparent communication [81]. Patients must evaluate what data they are comfortable disclosing, regardless of AI [82]. While the opinions about the extent of data sharing differ among participants, most Americans prefer to have control over the entire lifetime of their personal health data [17]. In line with our participants, they are also more likely to share data if the reason for doing so is plausible and the entities receiving the data appear trustworthy, e.g. physicians.

Our results can be compared across Germany, partly because we examined the patients' perceptions in two regions of Germany (Southwest and Mid‐East), and partly because existing German studies in other healthcare settings have reported similar results. Lennartz et al. showed, among other things, that patients would follow the physician's diagnosis in the case of different diagnoses [25]. Fritsch et al. found that patients are generally open to the use of AI and believe that physicians are responsible for the final decision [26]. Both studies were conducted in a clinical setting in Western Germany. Our findings are consistent with theirs, suggesting that patients' perceptions are broadly similar across regions of Germany and healthcare settings.

### 4.1 Strengths and Limitations

This study provides valuable insights into patients' perceptions of AI in medical care, with a diverse sample including outpatients as well as older, chronically ill, and socioeconomically disadvantaged individuals. However, the recruitment process as well as the examples and questions provided may have introduced bias, although the questions allowed for open‐ended responses and the examples helped to contextualise a mostly abstract topic. Nevertheless, the participants predominantly discussed hypothetical scenarios as most of them had no previous experience with AI in medicine.

### 4.2 Recommendations for Future Research

Future research should include participants from diverse ethnic backgrounds and from different healthcare settings like care recipients. It is also important to examine patients' perceptions on realistic scenarios before and after AI use, especially including applications for chronic disease management and preventive medicine, as well as exploring emerging subthemes. For practical use, the perceptions of other healthcare stakeholders affected by AI implementation, such as nurses and physicians, also need to be explored.

## 5 Conclusions

Patients could generally imagine AI as a support tool to assist medical staff, provided that the human relationship and the patient's well‐being are maintained. They considered that medical care can benefit from AI, particularly in terms of effectiveness and accuracy, which can be enriching in the collaboration between humans and AI. Adequate consideration of patient's needs, protection of patient data, transparency in data use, and tailored communication are crucial in the development and implementation of AI systems in healthcare. Furthermore, responsibilities for AI should be addressed to enable its practical use. Especially the perspectives of included outpatients, older, chronically ill and socioeconomically disadvantaged patients, and the resulting recommendations for AI development and implementation, add new insights to the current literature. Incorporating the considerations outlined in this study into the development of AI systems may improve the practical use of real‐life applications.

## Author Contributions

**Jana Gundlack:** investigation, methodology, formal analysis, visualisation, writing – original draft, writing – review and editing. **Sarah Negash:** project administration, investigation, formal analysis, methodology, writing – review and editing. **Carolin Thiel:** methodology, writing – review and editing. **Charlotte Buch:** methodology, formal analysis, writing – review and editing. **Jan Schildmann:** methodology, writing – review and editing. **Susanne Unverzagt:** supervision, writing – review and editing. **Rafael Mikolajczyk:** conceptualisation, funding acquisition, supervision, writing – review and editing. **Thomas Frese:** supervision, resources, writing – review and editing.

## Ethics Statement

The Ethics Committee of the Faculty of Medicine, Martin Luther University Halle‐Wittenberg, approved the study (protocol code 2021‐229).

## Consent

Participants were informed about the study and given the option to revoke their consent at any time without providing reasons or any disadvantages. All participants signed an informed consent form.

## Conflicts of Interest

The authors declare no conflicts of interest.

## Supporting information

Supporting information.

## Data Availability

The data that support the findings of this study are available on request from the corresponding author. The data are not publicly available due to privacy or ethical restrictions.
