# Supplementary material for: Artificial Intelligence in Medical Care – Patients' Perceptions on Caregiving Relationships and Ethics: A Qualitative Study
Source: Health Expect. 2025 Mar 17;28(2):e70216. doi: 10.1111/hex.70216 (PMC11911933; doi:10.1111/hex.70216)
Supplement: Supplementary file 1 — Supporting information. [file HEX-28-e70216-s001.docx]

## Supplement

**Supplementary file 1: Topic Guide**

**The themes and subthemes of the topic guide, as well as the questions posed to the participants (if not already answered in the discussion).**

(Referring to methods)

Table 1 Topic Guide

|  | **Topic** | **Questions** | **Remarks** | |
| --- | --- | --- | --- | --- |
| 1 |  | What comes to mind when you hear Artificial Intelligence? |  | |
|  | **Short video with definition of AI and digital application examples** | | |  |
|  | **Main Part** | | |  |
| 2 |  | Have you already had experience with AI systems in the medical field? Please explain your answer in more detail. |  | |
| 3 | **Advantages** | With the definition and examples in mind, what advantages/benefits do you think AI systems have in medical care? | Questioning different perspectives  - for patients  - for physicians | |
| 4 | **Disadvantages** | In your opinion, what disadvantages/risks do AI systems have in medical care? | Questioning different perspectives  - for patients  - for physicians | |
| 5 | **Subthemes** |  | If not already mentioned | |
| 6 | Discrimination | Please think back to the definition from earlier, where it was shown that AI collects, processes, and learns from data in order to perform certain tasks. What comes to your mind in this regard when it comes to equal or unequal treatment of patients? |  | |
| 7 | Privacy | What do you think about privacy in the context of AI in medical care? |  | |
| 8 | Comprehensibility | How important is it for you to be able to understand how an AI system works (eg, how the AI makes decisions)?  How important is it for you that your doctor can understand how an AI system works? Please give reasons for your statements. |  | |
| 9 | Responsibility | When you think of patients, physicians, and the developers of an AI system - where do you see the responsibilities of each party (in relation to AI)? |  | |
| **10** | **Acceptance** | What factors would make you more likely to accept an AI system?  What factors would make you less likely to accept an AI system?  What challenges do you see for a successful use (of AI in medical care)? | How do you think possible difficulties can be overcome? | |
| **11** | **Influence on patient-physician-relationship** | Please imagine that your treating doctor is supported by an AI system as part of your medical care. How does this affect your relationship with your treating physician?  Now imagine, for example, that the AI makes a diagnosis or treatment suggestion that differs from your physician's opinion. How do you deal with this? How should your doctor deal with this?   - Who would you trust more, the AI or your doctor? |  | |
| **12** | **Influence on patients** | How could the use of AI in medical care affect you as a patient? |  | |
| 13 | **Use** | Where do you see possible areas of application for AI in medical care, now or in the future?  Where do you tend not to see any areas of application for AI? Please give reasons for your statements. |  | |
|  | **Closing + Summary** | | |  |
| 14 |  | Is there anything else that has not been mentioned about today's topic? Anything else you would like to share with the group? |  | |

**Supplementary file 2: Characteristics of Focus Groups**

**The characteristics of the participants per focus group.**

(Referring to results)

Table 2 Characteristics of Focus Groups

| Variable | FG | FG 1 | FG 2 | FG 3 | FG 4 | FG 5 |
| --- | --- | --- | --- | --- | --- | --- |
| *Number of participants* | 5 | 5 | 5 | 6 | 8 | 6 |
| *Age, Median (Range), y* | 46.0  (37-55) | 71.0  (62-74) | 72.0  (41-92) | 36.5  (23-78) | 30.0  (24-56) | 40.5  (28-68) |
| *Gender* |  |  |  |  |  |  |
| Female | 3 | 1 | 2 | 3 | 3 | 1 |
| Male | 2 | 4 | 3 | 3 | 5 | 5 |
| *Highest education level* |  |  |  |  |  |  |
| General qualification for university entrance (12-13y) | 1 | 4 | 1 | 5 | 7 | 2 |
| General certificate of secondary education (9-10 y) ^a^ | 4 | 1 | 4 | 1 | 1 | 4 |
| *Vocational qualification ^b^* |  |  |  |  |  |  |
| Completed vocational training | 4 | 4 | 4 | 1 | 4 | 4 |
| In vocational training | NA | NA | NA | NA | 2 | NA |
| Advanced technical college certificate/ university degree | 1 | 4 | 1 | 5 | 4 | NA |
| No vocational qualification | NA | NA | NA | NA | NA | 2 |
| Other vocational qualification | NA | NA | 1 | 1 | NA | NA |
| *Employment status* |  |  |  |  |  |  |
| Employed | 5 | 1 | 1 | 4 ^c,d^ | 7 ^c^ | 1 |
| Not employed  thereof pensioners  thereof students | NA  NA | 4  4 | 4  4 | 1  1  1 ^e^ | NA  NA | 5  1  1 |
| In vocational training | NA | NA | NA | 1 | 2 | NA |
| *Chronic disease(s)* |  |  | ^f^ |  |  |  |
| Yes | 2 | 4 | 2 | 2 | 4 | 6 |
| No | 3 | 1 | 2 | 4 | 4 | NA |
| *Frequency of GP consultation* |  |  | ^f^ |  |  |  |
| Less than once every 3 months | 4 | 1 | 1 | 3 | 5 | 1 |
| Once every 3 months | 1 | 3 | 3 | 2 | 2 | 2 |
| 2 to 3 times in 3 months | NA | 1 | NA | 1 | NA | 2 |
| 4 or more times in 3 months | NA | NA | NA | NA | 1 | 1 |
| *Relationship to GP* |  |  |  |  |  |  |
| Very good | 3 | 4 | 3 | 3 | 3 | 2 |
| Rather good | 2 | 1 | 1 | 3 | 4 | 2 |
| Neutral | NA | NA | NA | NA | 1 |  |
| Rather poor | NA | NA | 1 | NA | NA | 1 |
| Very poor | NA | NA | NA | NA | NA | 1 |
| *Affinity for new technology*  Median and range ^g^ in FGs | 3.0  (2-4) | 2.67  (2-4.33) | 3.0  (2-4.33) | 3.67  (3-5) | 4.83  (2-5) | 4.17  (1-5) |
| ^a^ Includes the German “Hauptschulabschluss”  ^b^ Partially more than one vocational qualification existing  ^c^ One participant simultaneously in vocational training  ^d^ One participant simultaneously in retirement  ^e^ Participant additionally stated *others* for employment status  ^f^ One participant did not respond  ^g^ Scale: ≤ 2 low, > 2 to < 4 medium, ≥ 4 high  Abbreviations: FG - focus group, GP - general practitioner, y - years, NA - not applicable | | | | | | |
